# Brain Network Activation Technology Does Not Assist with Concussion Diagnosis and Return to Play in Football Athletes

**DOI:** 10.3389/fneur.2017.00252

**Published:** 2017-06-06

**Authors:** Steven P. Broglio, Richelle Williams, Andrew Lapointe, Ashley Rettmann, Brandon Moore, Sean K. Meehan, James T. Eckner

**Affiliations:** ^1^NeuroTrauma Research Laboratory, University of Michigan Injury Center, University of Michigan, Ann Arbor, MI, United States; ^2^NeuroTrauma Research Laboratory, University of Michigan, Ann Arbor, MI, United States; ^3^Michigan NeuroSport, University of Michigan, Ann Arbor, MI, United States; ^4^Human Sensorimotor Laboratory, University of Michigan, Ann Arbor, MI, United States; ^5^Department of Physical Medicine and Rehabilitation, Michigan NeuroSport, University of Michigan, Ann Arbor, MI, United States

**Keywords:** concussion, cognitive function, neuroelectric function, brain networking, neurostatus

## Abstract

**Background:**

Concussion diagnosis and management remains a largely subjective process. This investigation sought to evaluate the utility of a novel neuroelectric measure for concussion diagnosis and return to play decision-making.

**Hypothesis:**

Brain Network Activation (BNA) scores obtained within 72-h of injury will be lower than the athlete’s preseason evaluation and that of a matched control athlete; and the BNA will demonstrate ongoing declines at the return to play and post-season time points, while standard measures will have returned to pre-injury and control athlete levels.

**Design:**

Case–control study.

**Methods:**

Football athletes with a diagnosed concussion (*n* = 8) and matched control football athletes (*n* = 8) completed a preseason evaluation of cognitive (i.e., Cogstate Computerized Cognitive Assessment Tool) and neuroelectric function (i.e., BNA), clinical reaction time, SCAT3 self-reported symptoms, and quality of life (i.e., Health Behavior Inventory and Satisfaction with Life Scale). Following a diagnosed concussion, injured and control athletes completed post-injury evaluations within 72-h, once asymptomatic, and at the conclusion of the football season.

**Results:**

Case analysis of the neuroelectric assessment failed to provide improved diagnostics beyond traditional clinical measures. Statistical analyses indicated significant BNA improvements in the concussed and control groups from baseline to the asymptomatic timepoint.

**Conclusion:**

With additional attention being placed on rapid and accurate concussion diagnostics and return to play decision-making, the addition of a novel neuroelectric assessment does not appear to provide additional clinical benefit at this time. Clinicians should continue to follow the recommendations for the clinical management of concussion with the assessment of the symptom, cognitive, and motor control domains.

## Introduction

Despite decades of research, the concussion diagnosis and return to play decision remain clinical ones supported by objective measures such as neurocognitive testing, postural control, and subjectively reported symptoms (1–4). Individually, each measure offers sensitivity to acute post-injury changes in function that range around 60% (5, 6). When return to play is being considered, cognitive and balance measures have been shown to have less than optimal test stability (7–10) that negatively influences their usefulness and the medical staff must rely on the concussed athlete to honestly report symptoms. With growing public attention on concussion diagnosis and management, there is an increasing need to develop and validate objective measures that will limit the subjective component of the process and aid with clinical management.

Previous works related to concussion diagnosis have evaluated potential concussion biomarkers including functional magnetic resonance imaging (fMRI), diffusion tensor imaging (DTI) (11, 12), head impact biomechanics (13), and serum biomarkers (14). None of these measures have proven useful in the athletic environment. fMRI and DTI studies have yielded inconsistent post-injury findings [see Chamard et al. (15) and Dimou and Lagopoulos (16) for reviews]. Head impact biomechanics studies have failed to demonstrate a consistent biomechanical threshold for injury prediction (17), and the dynamics of serum biomarkers appear to be too slow for reliable sideline or post-session implementation (14). Similarly, implementation of these same serum biomarkers at the return to play time point has failed to demonstrate clinical utility for many of the same reasons (18). Furthermore, fMRI, DTI, and serum biomarkers have been shown to undergo ongoing changes that do not correlate with other standard clinical measures, bringing their utility into further question [see Henry (19) for review].

One potential assessment tool, used in various neurological conditions, that has received relatively little attention in concussion is electroencephalography (EEG). EEG is a non-invasive methodology used to monitor the brain’s electrical activity from the scalp. The recorded voltage fluctuations are thought to represent postsynaptic potentials associated with cerebral activity. More germane to concussion research has been the implementation of event-related potentials (ERPs), which reflect the phase-locked responses of neural generators at a single electrode on the scalp in preparation for or in response to a standardized event. Most recently, ERPs were implemented in soccer athletes with a diagnosed concussion within 24 h of injury. Compared to the non-contact athlete controls, the concussed athletes demonstrated deficits in attentional orienting and resource allocation (20). Additionally, ERPs have also been used to demonstrate ongoing changes in brain activity after the athlete has returned to baseline levels of clinical functioning [see Ref. (21) for review]. Phase-locked ERPs do not wholly quantify event-related brain dynamics as perturbations of the EEG signal that may contain equally relevant information. Instead, decomposition of the EEG signal into the various components that sum together to provide the signal measured by the surface electrode may provide additional insight into event-related neural responses. EEG recordings are commonly divided into the delta, theta, alpha, beta, and gamma bands (22), based on signal frequency and are associated with continuous tracking, inhibition, inhibition control, focus, and short-term memory, respectively (23). One study implemented EEG assessments within 24 h of injury and demonstrated altered waveforms, while also demonstrating ongoing EEG alterations 45 days post-injury, despite a return to clinically normal status (24).

The Brain Network Activation (BNA; ElMindA, Ltd., Herzliya, Israel) score was developed to capture the ability of the brain to use multiple anatomical regions during a cognitive task by encapsulating multiple scalp locations, frequency bands, amplitude, and timing of ERP signals collectively, rather than independently, in response to an event (25–29). Previous concussion works with BNA have established reliable change guidelines relative to pre-injury levels (30) and demonstrated the ability of BNA scores to differentiate between those with and without posttraumatic migraine symptoms following concussion (31). Furthermore, other reports have directly implemented BNA testing following concussion. Variable results were noted when BNA was applied post-concussion with one investigation demonstrating the ability to differentiate between concussed athletes and healthy matched controls (29) while another did not (32). Importantly, neither investigation implemented individualized pre-injury assessments for comparison to post-injury tests as recommended (1, 2).

The intent of this investigation is, therefore, to implement the BNA alongside standard clinical measures to evaluate its utility as for injury diagnosis and return to play decision-making among concussed athletes. Based on previous ERP and BNA works, we hypothesize BNA scores obtained within 72-h of injury will be lower than the athlete’s preseason evaluation and that of a matched control athlete. Furthermore, we hypothesize the BNA will continue to demonstrate ongoing declines with respect to baseline at the return to play and post-season time points, while standard measures will have returned to pre-injury and control athlete levels.

## Materials and Methods

As part of a larger investigation on the effects of repeated head impacts and cognitive functioning in varsity high school football athletes, 64 football athlete participants from a single high school were enrolled between the 2013 and 2015 football seasons. Prior to data collection, each athlete provided written assent and their parent/guardian provided written consent for this Institutional Review Board approved study. At the time of enrollment, the athletes were 15.9 (0.8) years, 179.5 (6.6) cm, 79.8 (14.1) kg, and reported 0.4 (0.7) previously diagnosed concussions.

Prior to the competitive season, each athlete completed a baseline evaluation that included the Cogstate Computerized Cognitive Assessment Tool (CCAT), the SCAT3 self-reported symptom checklist, quality of life with the Satisfaction with Life (SWL) and Health Behavior Inventory (HBI) scales, a clinical test of reaction time, and a BNA assessment utilizing an auditory oddball task. Each item is described below.

Throughout the football season, an athletic trainer was present at all games and practices. In the event, an athlete sustained a blow that resulted in a suspected concussion, and the athlete was evaluated following the recommended clinical guidelines (2). When the assessment indicated a concussion, the athlete was referred to a neurologist or primary care physician who made the concussion diagnosis. Nine concussions were confirmed during 3-year study period.

Once the concussion was diagnosed, the injured athlete and a matched control from the same team (based on playing status, primary position, and age) completed identical reassessments while still symptomatic within 72-h of injury, when the concussed athlete was declared asymptomatic and began a graded return to play progression, and at the end of the season.

### Assessment Measures

The Cogstate CCAT is a 15–20 min, computer-based assessment of cognitive function that asks the participant to respond to a series of digital playing cards and generates composite scores for processing speed, attention, composite learning, and working memory speed and accuracy. The Cogstate CCAT has demonstrated moderate reliability and sensitivity for concussion (33).

The SCAT3 symptom list includes 22 symptoms that are associated with acute sport concussion (34). The athlete was presented with the list and asked to indicate how he felt at that moment based on a 0 (none) to 6 (severe) Likert scale. The total symptom score were recorded for analysis. The symptom scale is applicable to those older than 13 (1) and has been reported to have moderate to high reliability and sensitivity (35).

The SWL scale is a 5-item scale developed to assess global life satisfaction in those as young as 7 years old (36). Each scale item is rated on a 7-point scale from strongly disagree to strongly agree with higher scores representing greater life satisfaction. A review of works using the SWL scale suggests it is sensitive to changes in life satisfaction (37) and maintains adequate reliability (36).

The HBI is a 20-item scale commonly used to evaluate prolonged concussion-related symptoms in the cognitive, somatic, emotional, and behavioral domains. Each symptom is graded on a four-point scale based on how the athlete felt over the previous seven days. Cognitive and somatic scores are generated with lower scores indicating more positive health behavior. The HBI has been demonstrated as an accurate assessment of concussion-related symptoms in a youth population (38).

The clinical reaction time test is a modified ruler drop test that employs a dowel rod affixed to a hockey puck and marked in 0.5 cm increments. With the athletes’ forearm resting on a table, the measuring stick is randomly dropped with the athlete grasping the rod as quickly as possible. The distance the device falls is converted into a reaction time. Two practice trials and eight testing trials were administered with the mean reaction time calculated over the eight testing trials (39). The clinical reaction time test has been shown to have moderate reliability (40) and moderate to high sensitivity among concussed athletes (41).

The BNA is an estimation of brain interconnectedness (i.e., networking) in response to an event and is calculated from electrophysiological data captured from a 256-channel Ag–AgCl wet lead surface electrode cap and 300 Amp amplifier (Electrical Geodesics, Inc., Eugene, OR, USA). Data were recorded at 256 Hz, band pass-filtered at 0.1–100 Hz, and stored offline for later BNA calculation. Eye blinks and motion were removed as outlined elsewhere (27). The electrophysiological data used to calculate the BNA score were recorded while the participant completed an auditory oddball task. This required the participant to listen to a series of tones with the frequent tone set at 2,000 Hz and occurring with 80% probability. The infrequent tone was set at 1,000 Hz and occurred with 10% probability. The oddball tone was a random noise (e.g., white noise, phone ring, knock on door, etc.) that occurred with 10% probability. The participant was instructed to disregard the frequent and oddball tones and respond to the infrequent tone by depressing a button held in the right hand. The stimuli were presented over a 16 min period.

A detailed explanation of the BNA algorithm has been reported elsewhere (31). Briefly, the process yields four output scores relating to amplitude, synchronization, timing, and connectivity that range from 0 to 100, with scores reflecting the relative similarity of the participant’s network to a pre-established reference network derived from a normative database. The work by Eckner et al. (30) provides a set of reliable change values for the BNA amplitude score calculated from the responses to the frequent tone to be used in the clinical interpretation of the data. No published reliable change intervals are available for the BNA synchronization, timing, and connectivity scores for the frequent tone or any BNA variable for the infrequent or oddball tones.

### Data Analysis

Data were not available for five of the nine concussed athletes who either did not immediately report their injury or were asymptomatic at the time of their initial after-injury reassessment. As a result, four cases are presented documenting changes in BNA scores while still symptomatic within 72-h of injury relative to baseline, asymptomatic, and end of season time points. Clinical interpretation of the case series results was based on guidelines provided by Kobau et al. (42) for the SWL scale, HBI guidelines from Yeates et al. (43), clinical reaction time values from Eckner et al. (41), SCAT3 symptoms by Chin et al. (44), CCAT scores from Nelson et al. (33) and Louey et al. (45), and BNA amplitude by Eckner et al. (30). Clinical interpretation guidelines for the BNA synchronization, timing, and connectivity scores are not available.

Statistical analyses for the baseline, asymptomatic, and end of season time points for the cohort of nine concussed and nine matched controls were completed as outlined below. One control participant was identified as an outlier using Grubbs’ test (46). Specifically, the participant showed significantly lower baseline and asymptomatic scores for synchronization (*G*_max_ = 2.55 and 3.03) relative to all other participants. Consequently, both the participant and his match were removed from subsequent analyses. Separate group × time (baseline, asymptomatic, and end of season) mixed-measures analysis of variance (ANOVA) was performed for each variable of interest. Time was treated as a repeated measure. Variables of interest included the CCAT composite scores (i.e., processing speed, attention, composite learning, and working memory speed and accuracy), SCAT3 total symptom severity, SWL total score, HBI cognitive and somatic scores, clinical reaction time in milliseconds, and BNA amplitude, synchronization, timing, and connectivity scores. Clinical performance on the oddball task was not available. Sphericity was evaluated with Mauchly’s test, and the Greenhouse–Geisser technique was implemented as indicated. *Post hoc* analyses were corrected using the Bonferroni method and effect sizes (Cohen’s *d*) were calculated. Finally, Pearson correlations were run between the BNA output scores and the traditional clinical measures implemented herein. An alpha level of 0.05 was used for all statistical tests performed using SPSS v22. Values presented in text are reported as mean ± SD unless otherwise denoted.

## Results

The eight concussed athletes (16.6 ± 0.5 years, 179.7 ± 6.9 cm, 83.3 ± 17.0 kg, 0.8 ± 0.7 previously diagnosed concussions) included in the analysis were matched to eight control athletes (16.6 ± 0.5 years, 180.7 ± 7.1 cm, 82.9 ± 16.3 kg, 0.4 ± 0.7 previously diagnosed concussions). There were no significant differences in demographic variables between groups (*p*’s > 0.05). Outcome data were available for all 16 participants at baseline, 4 concussed and 4 controls within 72-h of injury, all 16 participants at the asymptomatic and post-season time points.

### Case Series

**Figure 1:**
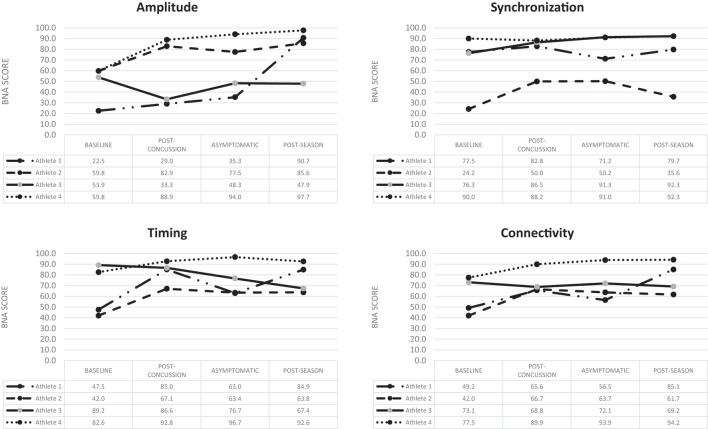
Change over time for the four BNA scores generated for the four concussed athletes evaluated at all four time points.

Athlete 1 completed his first post-injury evaluation within 24 h, and he was declared asymptomatic 4 days post-injury. Post-season testing was completed 52 days following the injury. BNA analysis showed an overall improvement over the four assessments for amplitude, synchronization, timing, and connectivity (Figure 1). Clinical measures showed the SWL fell within the high to average range (24–27) at all points and no meaningful change from baseline for the HBI (somatic or cognitive). Relative to baseline, reaction time increased to a clinically meaning level at the 72-h (+22 ms), asymptomatic (+33 ms), and post-season (+11 ms) time points. Total symptom severity increased by 13 at the 72-h assessment, but then returned to within normal range at the asymptomatic and post-season assessments. Performance on the CCAT indicated no clinically meaningful decline on any composite score.

Athlete 2 completed his first post-injury evaluation within 48 h, and he was declared asymptomatic 9 days post-injury. Post-season testing was completed 45 days after following the injury. The BNA analysis showed an overall improvement over the four assessments for amplitude, synchronization, timing, and connectivity (Figure 1). Clinical measures showed the SWL fell within the very high/highly satisfied range (34–35) at all points. The athlete did have a clinically meaningful increase on his HBI cognitive (+2) assessment at the initial post-injury evaluation that returned to baseline levels at the asymptomatic and post-season evaluations. The HBI somatic increased by 12 at the initial post-injury evaluation and was 3 higher than baseline at the asymptomatic point. He had returned to baseline at the post-season assessment. The reaction time assessment showed no clinically meaningful decline at any post-injury point, while total symptom severity increased by 27 at the 72-h assessment, but then returned to within normal range at the asymptomatic and post-season assessments. Performance on the CCAT indicated no clinically meaningful decline on any composite score.

Athlete 3 completed his first post-injury evaluation within 48 h, and he was declared asymptomatic 4 days after the injury. Post-season testing was completed 46 days following the injury. The BNA analysis showed clinically meaningful decline in amplitude (60% confidence interval) at the immediate post-injury assessment that return to pre-injury levels at the asymptomatic and post-season points. Synchronization showed an upward trend across the time points, while connectivity remained flat and timing showing an overall downward trend (Figure 1). Clinical measures showed the SWL fell within the very high/highly satisfied range (30–35) at all points. The athlete showed no change on his HBI cognitive assessment at all three post-injury assessments, but did report a clinically meaningful increase on his HBI somatic assessment at the initial post-injury (+6), asymptomatic (+6), and post-season (+7) evaluations relative to his baseline. The reaction time assessment showed a large increase in response time at the immediate post-injury (+38 ms) and post-season (+39 ms) evaluations, but the asymptomatic assessment was within 1 ms of the baseline. Total symptom severity increase by 8 at the immediate post-injury evaluation, but had returned to baseline levels at the asymptomatic and post-season time points. Performance on the CCAT indicated no clinically meaningful decline on any composite score.

Athlete 4 completed his first post-injury evaluation at 72-h, and he was declared asymptomatic 4 days after the injury. Post-season testing was completed 54 days after following the injury. The BNA scores remained flat or improved over the four assessments (Figure 1). Clinical measure showed the SWL fell within the very high/highly satisfied range (31–33) at all points and the athlete reported no meaningful increase in HBI cognitive or somatic scores. Clinical reaction time was slightly slower at the immediate post-injury evaluation, but returned to pre-injury levels at the asymptomatic and post-season evaluations. The athlete did have a meaningful increase in total symptom severity (+4) at the immediate post-injury evaluation, but the asymptomatic and post-season evaluations were similar to baseline. Performance on the CCAT indicated no clinically meaningful decline on any composite score.

### Statistical Results

**Figure 2:**
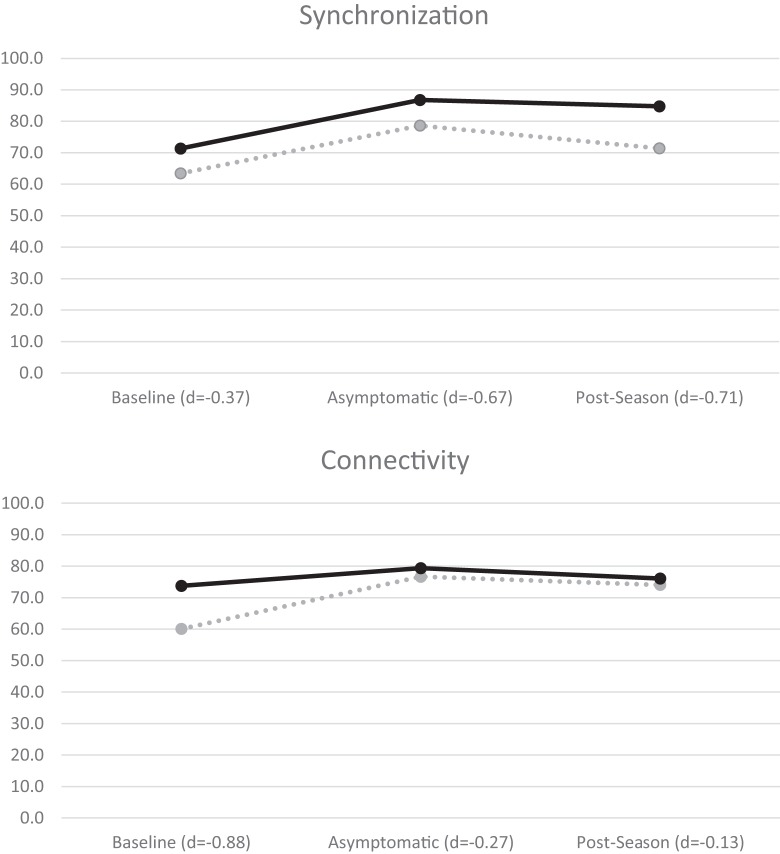
BNA synchronization (top) and connectivity (bottom) mean scores for all athletes. Concussed athletes are indicated with dotted lines and control athletes with solid lines. Negative effect sizes (Cohen’s *d*) indicate lower BNA scores by the concussed group.

A mixed-measures factorial ANOVA evaluated group (concussed/controls) differences in each of the four BNA scores outlined above over three time points (baseline, asymptomatic, and post-season) using recommendations from Maxwell and Delaney (47). A significant time effect for synchronization, *F*(2,22) = 6.56, *p* = 0.006, was found and *post hoc* contrasts revealed that both groups showed lower BNA scores at baseline in comparison to both asymptomatic, *F*(1,12) = 8.09, *p* = 0.015, and post-season values [*F*(1,12) = 8.31, *p* = 0.014; Figure 2]. A similar significant time effect was found for connectivity, *F*(2,22) = 3.81, *p* = 0.038, with the baseline score again being significantly lower than both the asymptomatic, *F*(1,11) = 6.15, *p* = 0.031, and post-season values [*F*(1,11) = 4.83, *p* = 0.05; Figure 2]. One-way ANOVA of the amplitude scores revealed a significant baseline difference, *F*(1,14) = 6.45, *p* = 0.024, with the concussed group (57.44 ± 18.33) demonstrating a lower score than controls (79.44 ± 16.28). Caution is in order while interpreting this result given that the time × group interaction approached significance (*p* = 0.054). No significant differences were found for timing or Connectivity (*p*’s > 0.05).

Analysis of variance of the baseline, asymptomatic, and end of season time points on the CCAT assessment indicated significant time main effects for composite learning (*p* = 0.01) and working memory speed (*p* = 0.04). In both instances, the post-season evaluation was significantly higher than the baseline evaluation (*p*’s < 0.05), and there were no significant group or group × time interactions (*p*’s > 0.05). In addition, there were no significant effects (group, time, or group × time) for the CCAT variables of processing speed, attention, or working memory accuracy (*p*’s > 0.05). Likewise, the analyses of the SCAT3 total symptom severity, SWL total score, HBI cognitive and somatic scores, and clinical reaction time indicated no significant main effects for group, time, or group × time interactions (*p*’s > 0.05). CCAT, SCAT3, SWL, HBI, and clinical reaction data are available in the Data Sheet S1 in Supplementary Material.

Correlational analyses identified a significant relationship between at least one BNA score and one traditional measure in two comparisons at baseline, two comparisons at the post-injury time point, and one comparison at the post-season time point. The 5 significant relationships, out of 64 analyses at each time point, fell within the expected range for type I error and were deemed spurious.

## Discussion

This investigation sought to evaluate the utility of a novel measure of brain function (i.e., BNA) in assisting with the diagnosis and return to play management of concussed athletes. Based on the case series and statistical findings presented here, the results do not suggest additional benefit of BNA scores over existing clinical measures. Patient level analyses at the 72-h assessment point indicated only one concussed athlete had a clinically meaningful BNA decline (60% CI) while two control athletes also showed declines (80–90 and <60% CI’s) at the same time point. These changes remained present at the asymptomatic and post-season evaluations. While challenges in identifying and assessing concussed athletes in a timely manner precluded group level assessments at the immediate post-injury time point, the evaluations performed at the asymptomatic time point failed to identify the anticipated group level differences between the concussed and control athletes. Indeed, all significant findings were associated with improvements in both clinical measures, and the BNA scores among the concussed and control athletes across the season. This finding is consistent with the work of Reches et al. (29) who were unable to demonstrate group differences in BNA performance among all concussed and control athletes when evaluated within 2–10 and 7–19 days post-injury with symptom recovery over the same interval. Similar results were also reported by Broglio et al. (32) who were unable identify differences in BNA performance among concussed and control athletes at identical recovery intervals to those implemented here. These findings are counter to those of Kontos et al. (31) who demonstrated the ability of BNA to differentiate between those with and without posttraumatic migraine symptoms 1 week following injury. Notably, Kontos et al. implemented a visual task (i.e., Go/No-Go) in place of the auditory task used herein. The Go/No-Go task also provides more reliable BNA scores [as denoted by Eckner et al. (30)] in comparison to the oddball paradigm, potentially accounting for the difference in findings.

Importantly, this is the first cohort investigation to implement BNA technology in a prospective manner that captured premorbid performance. Failure by others to collect pre-injury data (29) limits the ability of the authors to account for individual pre-injury differences between the concussed and control groups noted here (see Figure 2). This supposition is supported by Nuwer et al.’s (48) review of acute qEEG in mTBI which concluded that group level analyses are less sensitive to post-injury changes compared to individualized baseline evaluations. This argument is highlighted by the limited differences seen in group level explorations by others (29, 32); however, case studies implementing the BNA have been able to show compelling trends in post-injury recovery (25, 29).

Of the four injured athletes who completed an immediate post-concussion evaluation, only athlete 3 showed a clinically meaningful decrease in the BNA amplitude score suggestive of a change in cerebral networking brought about by concussion. This change, however, fell within the 60% reliable change confidence interval for this BNA score, indicating a potentially large margin for error. This concern is highlighted by the two control athletes also demonstrating declines at the same time point with no reported concussive injury. The concussed athlete (i.e., athlete 3) also demonstrated a large increase in his HBI somatic score, clinical reaction time, and SCAT3 symptom severity score. As the existing clinical management recommendations for concussion supported by many organizations (1–4) advocate for the use of these more traditional measures, which appear to offer greater sensitivity for concussion, it is unclear whether the addition of BNA to the assessment battery offers sufficient gains in clinical utility to justify the associated time and expense. Indeed, previous work implementing measures of cognitive functioning following concussion demonstrated moderate to high sensitivity to injury (33), with the combination of cognitive functioning, postural control, and symptom reports yielding suitable sensitivity for clinical use (6, 49).

Finally, in our analysis, one control had to be removed given his presence as a clear outlier. As such the invariant model utilized in calculating the BNA score may not be as robust as previously reported (27). Moreover, it may not adequately account for group variability in smaller samples as stated by Stern et al. (50). This argument can be further assured when we consider that both papers discussing the utility of BNA in sports-related brain injury (25, 29) presented BNA data on selected cases from a larger sample, rather than group level analyses (25, 29).

In a similar line of research, quantitative EEG analysis among concussed and control high school athletes demonstrated significant changes in cerebral electrophysiology within 24 h of injury and at post-injury day 8, with resolution by day 45 (51). Nearly identical findings were reported in a similar cohort of high school and collegiate athletes (52). Likewise, another study in which EEG data were collected in 65 male college and high athletes within 24 h of concussion (24) demonstrated, using an EEG analysis with linear and non-linear features of the brain’s electrical activity to generate a traumatic brain injury index, that larger changes were present among those with longer recovery periods (≥14 days) relative to those with a shorter recovery time (>14 days). Further, Prichep et al. (53) successfully utilized brain electrical activity to categorize, with a high level of sensitivity, patients with a structural brain injury, concussion, and normal controls. By contrast, one previous study comparing BNA scores between athletes with acute concussion and matched controls failed to identify differences between the two groups (32).

The reason why expected electrophysiological changes associated with concussion were not identified by BNA in this study is unclear. One possibility is that the concussions sustained by participants in this study may not have been as severe as the concussions in those enrolled in other investigations. If this is the case, it is possible that through the natural recovery process, and any electrophysiological changes initially present could have normalized in three of the four concussed athletes by the time of their initial BNA assessments. It should be noted that while reliable change indices ranges are not available for the synchronization, timing, and connectivity BNA scores, the observed changes in these scores between the baseline and initial post-injury assessment time points were small and often positive.

### Limitations

The ability to draw conclusions from this relatively small case series is limited. With many studies demonstrating electrophysiological changes following concussion and preliminary evidence suggesting the ability of BNA scores to discriminate between those with and without and concussion (29) or posttraumatic migraine (31), additional work comparing post-concussion BNA scores to athletes’ own baseline in a larger sample is justified. Future works should be mindful of the potential for practice effects with a pre-/post-injury study design (54) and should consider implementation of other stimuli sets (e.g., Flanker task or visual odd-ball) that may prove more useful in this clinical population. In addition, since the time of data collection and analysis, an age-specific amplitude score and a new BNA measure for youth athletes are now available. How these new algorithms and scores influence the findings presented here is not clear, but should be implemented in future works.

## Conclusion

Over the previous decade, the attention and interest in sport concussion has grown considerably. The result has been an increased focus on the diagnostic and return to play decision-making process. As the current standard of care relies on the clinical judgment of the practitioner, sensitive tools are needed to reduce or eliminate the subjective component of the process. The BNA technology was developed as a potential objective concussion assessment tool, but the preliminary findings herein failed to show a capacity to identify clinically meaningful declines in cerebral networking in a small sample within 72-h of injury or ongoing declines at the asymptomatic time point. As such, this study does not support added clinical utility of BNA scores over the currently implemented multifaceted clinical protocol that includes measures cognitive functioning, self-reported symptoms, reaction time, and quality of life in the assessment of concussed high school football athletes.

## Ethics Statement

This study was carried out in accordance with the recommendations of University of Michigan Institutional Review Board with written informed consent from all subjects. All subjects gave written informed consent in accordance with the Declaration of Helsinki. The protocol was approved by the University of Michigan Institutional Review Board.

## Author Contributions

SB aided in the concept, design, data collection, data analytics, manuscript drafting, and final approval. RW aided in data collection and final approval. AL aided in data analytics, manuscript drafting, and final approval. AR and BM aided in data collection and final approval. SM and JE data analytics, manuscript drafting, and final approval.

## Conflict of Interest Statement

All authors declare that the research was conducted in the absence of any commercial or financial relationships that could be construed as a potential conflict of interest.
